# Effects of temperature and salinity on respiratory losses and the ratio of photosynthesis to respiration in representative Antarctic phytoplankton species

**DOI:** 10.1371/journal.pone.0224101

**Published:** 2019-10-21

**Authors:** Deborah Bozzato, Torsten Jakob, Christian Wilhelm

**Affiliations:** 1 University Leipzig, Institute of Biology, Plant Physiology, Leipzig, Germany; 2 Saxon Institute of Biotechnology, Leipzig, Germany; University of Nantes, FRANCE

## Abstract

The Southern Ocean (SO) is a net sink for atmospheric CO_2_ whereby the photosynthetic activity of phytoplankton and sequestration of organic carbon (biological pump) plays an important role. Global climate change will tremendously influence the dynamics of environmental conditions for the phytoplankton community, and the phytoplankton will have to acclimate to a combination of changes of e.g. water temperature, salinity, pH, and nutrient supply. The efficiency of the biological pump is not only determined by the photosynthetic activity but also by the extent of respiratory carbon losses of phytoplankton cells. Thus, the present study investigated the effect of different temperature and salinity combinations on the ratio of gross photosynthesis to respiration (rGP/R) in two representative phytoplankton species of the SO. In the comparison of phytoplankton grown at 1 and 4°C the rGP/R decreased from 11.5 to 7.7 in *Chaetoceros* sp., from 9.1 to 3.2 in *Phaeocystis antarctica* strain 109, and from 12.4 to 7.0 in *P*. *antarctica* strain 764, respectively. The decrease of rGP/R was primarily dependent on temperature whereas salinity was only of minor importance. Moreover, the different rGP/R at 1 and 4°C were caused by changes of temperature-dependent respiration rates but were independent of changes of photosynthetic rates. For further interpretation, net primary production (NPP) was calculated for different seasonal conditions in the SO with specific combinations of irradiance, temperature, and salinity. Whereas, maximum photosynthetic rates significantly correlated with calculated NPP under experimental ‘Spring’, ‘Summer’, and ‘Autumn’ conditions, there was no correlation between rGP/R and the respective values of NPP. The study revealed species-specific differences in the acclimation to temperature and salinity changes that could be linked to their different original habitats.

## Introduction

The Southern Ocean (SO) plays a pivotal role for Earth’s climate by controlling the amount of dissolved inorganic carbon stored in the ocean. The SO is considered as a net sink for atmospheric CO_2_ due to the cooling of southward directed subtropical surface waters which increases the solubility of CO_2_. This mechanism represents the so-called solubility pump whereby the majority of dissolved CO_2_ is sequestered in the deep ocean [1]. However, about 10% of the total amount of CO_2_ sequestration is assigned to the biological pump [2]. Accordingly, in the euphotic zone, phytoplankton cells photosynthetically assimilate inorganic carbon, which is then transferred as organic carbon to the deep ocean by sedimentation. The efficiency of this carbon transfer depends on the physical characteristics of the SO such as water temperature, extent of sea ice cover, wind speed, stratification, changes in nutrient dynamics, pH, light conditions, and salinity of surface waters ([3], reviewed in [4]). All these parameters will be altered by climate change and, as a consequence, will also influence the physiology (e.g. photosynthesis and respiration activity) and ecology (e.g. species composition) of phytoplankton in the SO [5,6]. The physiological response of phytoplankton cells to the expected changes in the SO’s physics is poorly understood [7,8] and represents a considerable gap of knowledge. For instance, it is not known how the balance between photosynthetic carbon assimilation and respiratory carbon losses depends on environmental conditions and seasonal changes. Although the data base to estimate the proportion of phytoplankton respiration to total microbial respiration is scarce, from the observed correlation of community respiration rates and chlorophyll concentrations it could be assumed that phytoplankton respiration contributes to a large part of community respiration at least in coastal waters of the SO [9] (and ref. therein). Since phytoplankton in the SO experience extreme variations in daily solar irradiance during the growth period due to changing day lengths (ranging from very few hours in winter to 20 hours in summer [10]) and sea ice cover extent, it could be assumed that respiratory losses have a stronger impact on the net primary production (NPP; equal to the difference between gross photosynthesis rate, GP, and respiration rate) in Antarctic waters than in temperate waters. Particularly, under short daylength or under deep-mixing conditions phytoplankton respiration strongly influences the algal biomass balance [11]. Unfortunately, there is a strong methodological limitation for the determination of phytoplankton respiration rates in natural habitats (e.g. distinction from heterotrophic respiration, limited comparability between alternative methods like O_2_ and ^14^C [12]) and only scarce information about respiration rates and the variability of the ratio gross photosynthesis to respiration (rGP/R) in phytoplankton of the SO. Nevertheless, studies have shown that rGP/R is indirectly correlated with temperature in phytoplankton from the SO [11,13]. Importantly, changes in rGP/R are primarily due to the fact that respiration rates are more temperature-dependent than photosynthetic rates [11]. It is known that respiratory losses in the euphotic zone of coastal waters range from 7 to 34% of GP and could reach even 50% of GP under bloom conditions with high chlorophyll concentrations [9,14]. Thus, the knowledge on the variability of respiratory losses and rGP/R is important for the evaluation of the carbon budget of the SO and particularly for the prediction of future development in the light of climate change. However, to our knowledge species-specific respiratory losses and the variability of rGP/R in phytoplankton from the SO were not investigated systematically under a combination of relevant different environmental factors (e.g. temperature and salinity).

In the present study, we investigated the physiological response of two Antarctic phytoplankton species in an experimental setup with combined changes of temperature and salinity. Accordingly, the diatom *Chaetoceros* sp. and two strains of the Haptophyte *Phaeocystis antarctica* were chosen as typical representatives of SO phytoplankton. Whereas *Chaetoceros* sp. shows a high abundance in sea ice and represents a dominating diatom species [15,16], *P*. *antarctica* is a typical pelagic phytoplankton species living in deeply mixed water [17,18]. Diatoms and Haptophytes differ in their light acclimation potential and show different physiological plasticity ([6] and ref. therein). For *P*. *antarctica*, two strains were investigated from very different geographic origins, namely from the Lazarev Sea and from Prydz Bay.

With respect to seasonal dynamics and the expected future changes in SO’s physics, the algae were cultivated under nine different combinations of salinity and temperature conditions. With this combination of phytoplankton species and experimental conditions it was intended to investigate the influence of temperature and salinity on rGP/R in general but also in the light of possible species-specific differences. In addition, the impact of changes in rGP/R on NPP under different seasonal conditions was evaluated.

## Material and methods

### Culture conditions

Cultures of the Antarctic diatom *Chaetoceros* sp. and two strains of the Haptophyte *Phaeocystis antarctica* were obtained from Dr. Steffi Gäbler-Schwarz (AWI Bremerhaven, Germany). The *Phaeocystis* strains were sampled and isolated on RV Polarstern cruises and at an Antarctic research station between 2005 and 2007 [19] whereby strains 109_27 and 764_48 were isolated from the Lazarev Sea (ANT XXIII-2) and from Prydz Bay (ANT XXIII-9), respectively. All cultures were grown in GP5 Medium [20] modified in this study with respect to the use of specific amounts of marine salt (Dupla Marin, Dohse Aquaristic, Koblenz, Germany) instead of seawater to yield the desired salinity of the medium (details below). The cultures were maintained in polystyrene culture flasks with filter screw caps (Carl Roth) in a climate chamber (Economic Lux Chamber, Snijders Labs) under low-light conditions (10 μmol photons m^-2^ s^-1^; 16:8 hours light-dark cycle). The cultures were used for experiments in their exponential growth period between 6 and 10 days post inoculation. The number of replicates (n) given in the results section is equivalent to the number of biological replicates (a detailed list of the number of replicates is presented in **S1 Table**). Since the measurements of oxygen evolution rates were characterized by a relatively low signal-to-noise ratio the number of biological replicates for this type of measurements was expanded up to n = 11 to enhance the statistical significance.

Three different temperature treatments were applied, namely -1°C, 1°C, and 4°C (± 0.5°C), in combination with different salinities of the growth medium: 20, 35, 50, and 70 practical salinity units (PSU; **S1 Table**). More precisely, growth temperature of -1°C was combined with salinities of 35, 50, and 70 PSU whereas growth temperatures of 1°C and 4°C were combined with salinities of 20, 35, and 50 PSU, respectively. The combinations of 20 PSU at -1°C and 70 PSU at 1 or 4°C were omitted since they are practically impossible. A salinity well below 35 PSU can be found only in regions with melting sea ice (T > 0°C) whereas salinities as high as 70 PSU can be reached only in the brine channels of sea ice (T < 0°C). The salinity of the medium was adjusted by the addition of the respective amount of marine salt. Depending on the growth rates of the cultures under the different experimental conditions, the cultures were acclimated for a period of at least two weeks (usually four weeks) to the new condition before starting physiological measurements.

### Chlorophyll a determination

Chlorophyll *a* (Chl*a*) concentrations were determined spectrophotometrically by extraction with 90% acetone according to the protocol from [21]. Algal samples (5 mL) were collected on glass-fiber filters, 2.5 mL acetone was added, and cells were broken in a cell homogenizer (Precellys Evolution, Bertin Technology, France). After centrifugation (2 min, 12.500 x *g*, Sigma 1–14, Sigma, Germany), absorbance of the pigment extract was measured with a spectrophotometer (Hitachi U2000, Tokyo, Japan) at 664 and 630 nm.

### Measurements of photosynthesis rates and variable chlorophyll fluorescence

Oxygen-based (P_O_) and fluorescence-based (P_F_) photosynthesis rates were measured and calculated as described in detail in [22]. Essentially, oxygen evolution and variable Chlorophyll (Chl) fluorescence were measured by light-irradiance curves (P-E curves) in a so-called Light pipette equipped with a special cuvette (Topgallant LLC, Salt Lake City, UT, USA) that allows the connection to a PAM-fluorometer (PAM 101/103, Walz, Effeltrich, Germany). A 3-ml aliquot of cells (equals a Chl*a* concentration of 4–6 μg mL^-1^) from each experimental condition was transferred into the cuvette and maintained at the respective growth temperature under continuous stirring in darkness for 5 min. For P-E curves, six actinic light levels (21, 50, 107, 207, 415, 713 μmol photons m^−2^ s^−1^) were applied for 4 min each. These light periods alternated with dark periods of 4 min length each. Measurements of P-E curves always started with an initial 4-min dark period yielding a total dark adaptation period of 9 min duration. Oxygen evolution was measured using a Clark-type electrode (MI 730, Microelectrodes Inc., NH, USA). For the calculation of P_O_ (μmol O_2_ [mg Chl*a*]^-1^ h^-1^) the oxygen solubility corrected for the medium salinity and the measuring temperature [23] was taken into account. Net oxygen evolution and dark respiration rates were derived from the average oxygen evolution rates measured during the last minute of each light and dark period, respectively. A representative example of light-dependent net oxygen evolution for *Chaetoceros* sp. and *P*. *antarctica* is shown in **S1 Fig**. Gross oxygen production was derived by correcting net oxygen evolution rates for the corresponding dark respiration (R; μmol O_2_ [mg Chl*a*]^-1^ h^-1^) measured after the respective light periods. It should be noted that no enhanced post-illumination respiration [24] was observed in the measurements. Moreover, the respiration rates showed very little variability with respect to the preceding irradiance levels.

The ratio of photosynthesis to respiration (rGP/R) was derived from the maximum value of fitted (details see below) gross photosynthesis (GP_max_) divided by the mean value of all respiration rates measured within a specific P-E curve.

In parallel with oxygen evolution, the variable Chl fluorescence parameters were determined, whereby Fo and Fm are the minimum and maximum fluorescence in darkness, respectively, and F and Fm’ are the steady-state minimum and maximum fluorescence under actinic illumination, respectively. Fluorescence-based photosynthetic rates (μmol O_2_ [mg Chl*a*]^-1^ h^-1^) were estimated as:

$$P_{F}=\Phi_{PSII}\times Q_{phar}\times 0.5\times 0.25/(d\times Chl)$$ (1)

where Φ_PSII_ is the effective quantum yield of PSII [25], Q_phar_ is the amount of absorbed radiation (see below), *d* is the optical path length of the measuring cuvette, and Chl is the Chl*a* concentration of the algal suspension. The factors 0.5 and 0.25 are based on the assumption that the linear transport of one electron requires two quanta and that four electrons are required for the evolution of one molecule of oxygen, respectively. It is thus assumed that P_F_ represents the maximum amount of electrons (expressed as oxygen equivalents) transported through the electron transport chain, whereas P_O_ is the oxygen evolution rate of PSII biased by alternative electron pathways, such as the Mehler-reaction or cyclic electron transport [26]. Therefore, the ratio P_F_/P_O_ describes the activity of alternative electron-consuming reactions [27,28]. A representative example of fluorescence-based P-E curve measured in *Chaetoceros* sp. and in *P*. *antarctica* is shown in **S1 Fig**.

The oxygen-based and fluorescence-based P-E curves were fitted according to [29]. The derived fitting parameters (a, b, and c) were used to calculate GP_max_ and the light saturation index (*E*_k_ value) according to [29]:

$${GP}_{\max}=1/(b+2\sqrt{a\times c})$$ (2)

$$E_{k}=c/(b+2\sqrt{a\times c})$$ (3)

In addition to the estimation of P_F_, the variable fluorescence parameters were used to calculate the extent of non-photochemical quenching (NPQ) according to [30]:

$$\mathrm{NPQ}=(Fm-Fm^{\prime })/Fm\prime$$ (4)

where Fm is the maximum fluorescence measured at the end of the initial dark period of P-E curve measurements. The maximum NPQ values (NPQ_max_) and the half-saturation irradiance of NPQ_max_ (*E*_50_) were derived from fitting of the light-response curves of NPQ using the Hill equation (according to [31]). A representative example of the fitted light-dependent NPQ measured in *C*. sp. and in *P*. *antarctica* is shown in **S1 Fig**.

### Cellular optical properties

The *in vivo*-absorption spectra of algal cells were measured in a dual-beam spectrophotometer (M500, Zeiss, Jena, Germany). The photometer was equipped with an adapter for dispersive samples (Zeiss) to allow a very close placement of the sample to the detector and to correct for light scattering. The Chl*a*-specific *in vivo*-absorption coefficient, a*_phy_ (cm^2^ [mg Chla]^-1^) was calculated as:

$$a_{phy}^{*}(\lambda )=2.3\times A(\lambda )/d\times \mathrm{Chl}$$ (5)

where 2.3 is the conversion factor from log10 to ln, *A* is the absorption of the sample (400–700 nm), *d* is the path length of the cuvette (0.01 m), and Chl is the Chl*a* concentration of the sample (mg m^-3^). In the results section, the mean values of the Chl-specific absorption (${\bar{a}}_{phy}^{*}$) are given.

The knowledge of the emission spectra of the light source and of a*_phy_ allows the estimation of the amount of photosynthetically active radiation absorbed by the algal cultures, *Q*_phar_. The estimation is based on the following equation (according to [32]):

$$Q_{\mathrm{phar}}=\int_{400\;nm}^{700\;nm}Q(\lambda )-Q(\lambda )\times e^{-(a_{\mathrm{phy}}^{*}(\lambda )\times \mathrm{Chl}\times d)}$$ (6)

where *Q*_phar_ is the photosynthetically absorbed radiation (μmol m^-2^ s^-1^), *Q* is the photosynthetically available (incident) radiation (μmol m^-2^ s^-1^), and *d* is the optical path length (m).

### Estimation of net primary production

To describe the potential effect of different rGP/R on NPP under different seasonal conditions (see below) the expected daily NPP was estimated from measured oxygen-based P-E curves (P_O_; see above) and considering the measured respiration rates. For the respective experimental conditions, the mean values of light-dependent GP (derived from measured P-E curves, see above) were fitted according to [29]. It should be noted that the fit function does not include a term for the initial respiration rate. Therefore, only P-E curves based on GP can be fitted in this way. The derived fitting parameters (a, b, c) were used to estimate daily NPP (μmol O_2_ [mg Chl*a*]^-1^ d^-1^) as:

$$NPP=\int_{0h}^{24h}(E/[(a\times E^{2})+(b\times E)+c])-R$$ (7)

where E is the amount of incident irradiance (μmol photons m^−2^ s^−1^; see below) and R is the respiration rate. The respiration rates were derived from the mean value of all respiration rates measured within a specific P-E curve. The incident irradiance was based on four daily light climates (**S2 Fig**) representing model estimates of different seasonal *in situ*-light conditions (adopted from [10]): winter sea ice, spring melt water, summer pelagic water, and autumn new sea ice. These light climates were combined with the fitting parameters derived from specific temperature and salinity conditions that reasonably represent the seasonal conditions during spring, summer, autumn, and winter (**Table 1**). To take into account the dynamics of light conditions, NPP was estimated for 10-min time intervals and integrated over 24 h.

**Table 1:** Experimental conditions and assumed light conditions used for the estimation of daily net primary production (NPP) under different seasonal conditions from measured photosynthesis and respiration rates. In case of a given range of temperature or salinity values, NPP was calculated as mean value of the respective NPP at the specific conditions. Light conditions were adopted from [10].

Season	Temperature	Salinity	Light condition
**Spring**	1°C	20 PSU	Meltwater
**Summer**	4°C	35 PSU	Pelagic
**Autumn**	1°C	35 PSU	New sea ice
**Winter**	-1°C	50 PSU	Sea ice

### Statistical analysis

Two-way analysis of variance (ANOVA) followed by Bonferroni post-tests (p-value < 0.05) were performed on the physiological data (GP_max_, R, rGP/R, NPQ_max_, P_F_/P_O_, a*_phy_) to test for differences of the algal species in response to culture conditions (temperature, salinity). The different salinity and temperature conditions were used as treatment factors. The data set was checked for normality by Shapiro-Wilk test (SigmaPlot 12.5), and all random samples passed the test. Correlation was calculated by Spearman rank correlation test (two-tailed test of significance with 95% confidence interval).

## Results

### Physiological key parameters

The data from P-E curves were used to compare the maximum gross photosynthesis rates (GP_max_), respiration rates (R), ratio of GP_max_ to respiration (rGP/R), NPQ_max_, and ratio of maximum fluorescence-based/maximum oxygen-based photosynthesis rates (P_F_/P_O_) for all experimental conditions and for the three algal strains used in this study (see below). With this wide set of experimental conditions it was intended to find general physiological responses of the investigated species to different temperature and salinity conditions. It has to be mentioned that at a growth temperature of -1°C the two strains of *P*. *antarctica* did not grow sufficiently well at 70 PSU to obtain sufficient biomass for physiological measurements. Therefore, under this temperature/salinity combination physiological measurements were performed for *Chaetoceros* sp. only. In addition to the determination of physiological parameters, data of P-E curves were also used to apply a curve fit according to [29] and to finally estimate the effects of changes in rGP/R on NPP for different environmental scenarios (see below).

**Fig 1:**
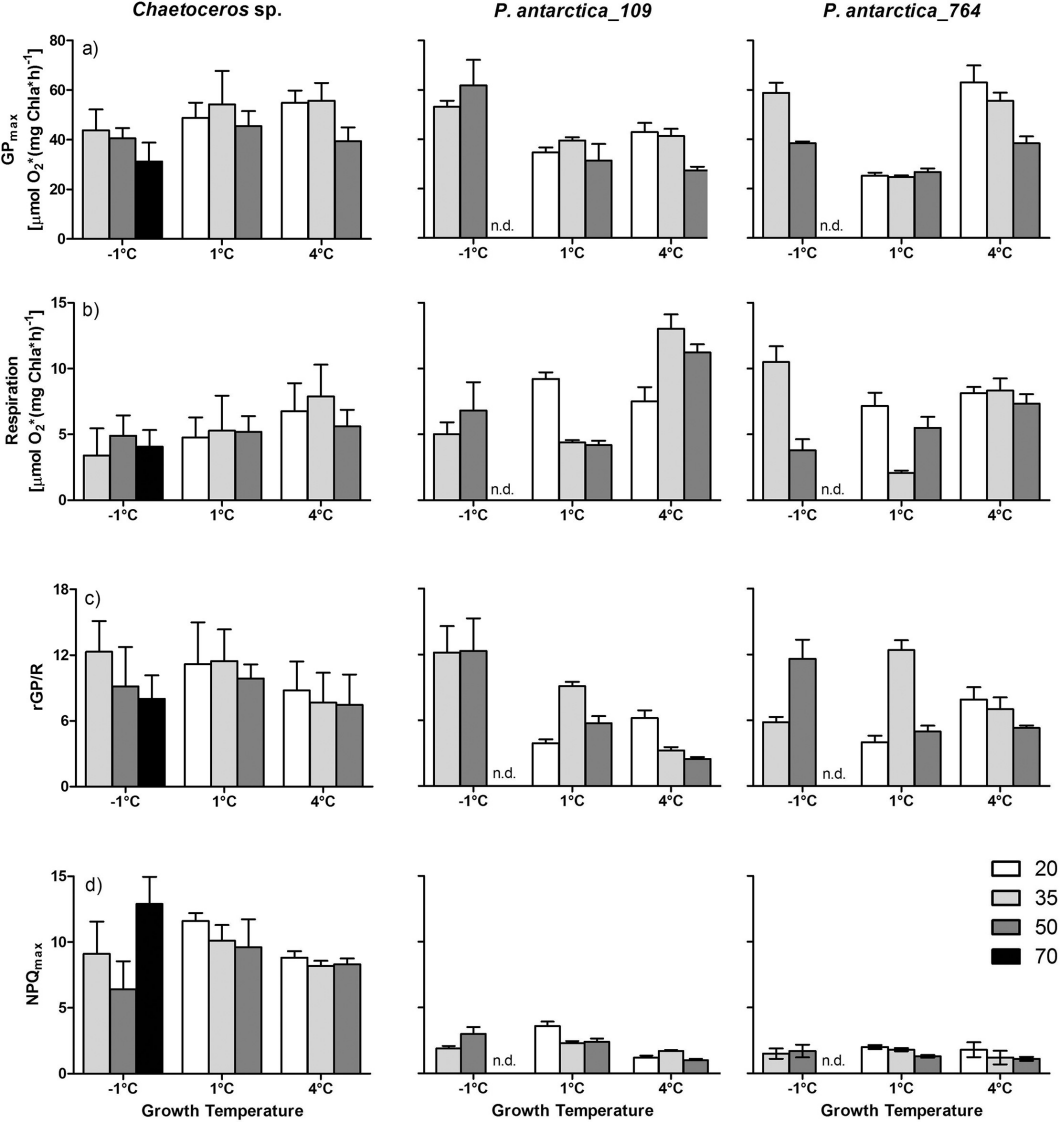
Physiological key parameters (GP_max_, R, rGP/R, NPQ_max_) of *Chaetoceros* sp. and *Phaeocystis antarctica*. Mean values (± standard deviation, n = 4–11) of physiological parameters measured in *Chaetoceros* sp. and *P*. *antarctica* (strains 109 and 764) grown under different combinations of temperature (-1, 1, 4°C) and salinity of the growth medium (20, 35, 50, 70 PSU; white, light grey, dark grey, black bars, respectively): a) Maximum gross oxygen-based photosynthesis (GP_max_, [μmol O_2_ (mg Chl*a*)^-1^ h^-1^]), b) Respiration rate (R, [μmol O_2_ (mg Chl*a*)^-1^ h^-1^]), c) Ratio of maximum gross photosynthesis rate to respiration rate (rGP/R), d) Maximum value of non-photochemical quenching (NPQ_max_), ‘n’ depicts the number of biological replicates. For *P*. *antarctica* no data were obtained at the condition -1°C/70 PSU (marked with ‘n.d.’).

**Fig 1A** shows the mean values of GP_max_ (Gross oxygen-based photosynthesis) at three different growth temperatures and in combination with different salinities. For *Chaetoceros* sp. no significant effect of temperature on GP_max_ was observed which is in contrast to *P*. *antarctica*. At the salinities 35 and 50 PSU, *P*. *antarctica* strain 109 showed significantly higher GP_max_ at -1°C than at 1°C (p < 0.001) whereas no temperature effect was detected for the comparison of GP_max_ measured at 1 and 4°C. For *P*. *antarctica* strain 764, a comparable increase of GP_max_ from 1°C to -1°C (p < 0.001) was found for 35 PSU, only. Moreover, only for *P*. *antarctica* strain 764 a significant increase of GP_max_ from 1°C to 4°C was observed at 20 and 35 PSU (p < 0.001). For all tested species an influence of salinity on GP_max_ was observed at 4°C with significantly lower GP_max_ values at 50 PSU than at 20 PSU (p < 0.01) and 35 PSU (p < 0.05), respectively. Significant species-specific differences were found at a growth temperature of 1°C with significantly higher GP_max_ values in *Chaetoceros* sp. (p < 0.01) than in both strains of *P*. *antarctica*. Significant differences in GP_max_ in comparison of strain 109 and 764 of *P*. *antarctica* were observed at 4°C (p < 0.05) only.

**Fig 1B** depicts the respiration rates under the applied experimental conditions. In *Chaetoceros* sp., a trend of increasing respiration rates with temperature was found at a salinity of 35 PSU with significant differences between -1 and 4°C (p < 0.001). In *P*. *antarctica* strain 109 a comparable effect was observed at 35 and 50 PSU with a significant increase of respiration rates at 4°C compared to 1°C (p < 0.01) and at 4°C compared to -1°C (p < 0.01). In *P*. *antarctica* strain 764 a significant increase of respiration rates with temperature was observed only at a salinity of 35 PSU in the comparison of 4 to 1°C (p < 0.001). In the comparison of the different species the most prominent result is the significantly higher respiration rate at 4°C/35 and 50 PSU in *P*. *antarctica* strain 109 compared to strain 764 (p < 0.01) and to *Chaetoceros* sp. (p < 0.001).

**Fig 2:**
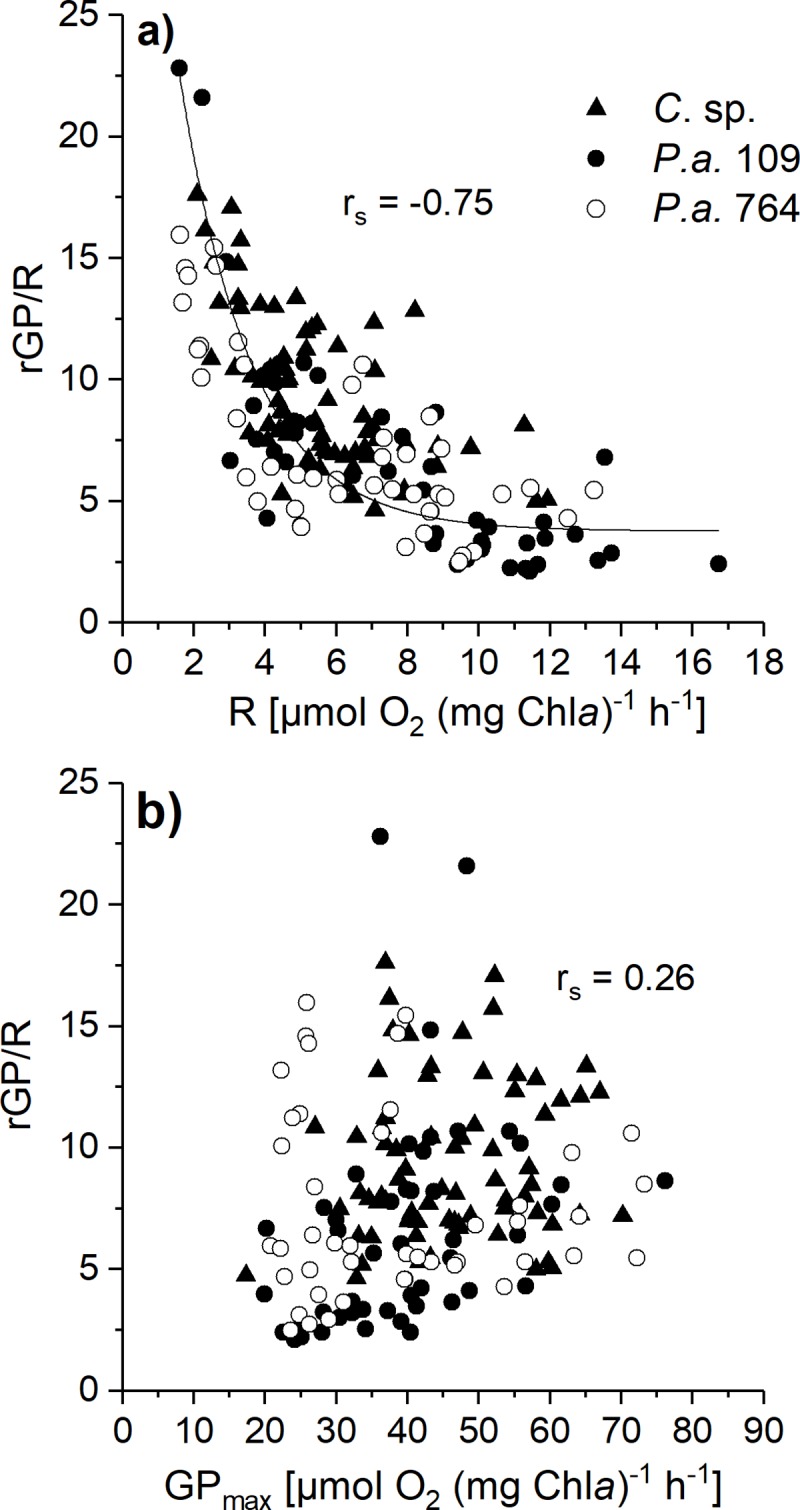
Relationship between a) ratio of gross photosynthesis to respiration (rGP/R) and respiration and b) rGP/R and maximum gross photosynthetic rates (GP_max_) in *Chaetoceros* sp. and *Phaeocystis antarctica* (strains 109 and 764). Cultures of *Chaetoceros* sp. (filled triangles), *P*. *antarctica* strain 109 (filled circles), and strain 764 (open circles) were grown under different combinations of temperature (-1, 1, 4°C) and salinity of the growth medium (20, 35, 50 PSU). The correlation was calculated using Spearman rank correlation (correlation coefficient r_s_).

**Fig 1C** depicts the ratio GP_max_ over respiration (rGP/R). At first sight, the temperature- and salinity-induced changes of GP and R seem to influence the ratio GP/R rather randomly. However, a few general trends could be deduced. Accordingly, for all investigated species rGP/R decreased from 1°C to 4°C at 35 PSU (p < 0.05). This trend of lower rGP/R with increasing temperature was measured in *Chaetoceros* sp. and *P*. *antarctica* strain 109 also in the comparison of -1 to 4°C (at 35 PSU; p < 0.01). Salinity was of minor importance on changes in rGP/R. In both strains of *P*. *antarctica*, only at a growth temperature of 1°C a significantly higher rGP/R was observed at 35 PSU compared to 20 and 50 PSU, respectively (p < 0.01). Significant species-specific differences were detected particularly at 1°C with higher rGP/R in *Chaetoceros* sp. than in both strains of *P*. *antarctica* (at 20 and 50 PSU; p < 0.05). Another important general trend was found in the relation of rGP/R to R and GP_max_, respectively. Whereas rGP/R significantly correlated to changes in respiration rates (p < 0.01) there was no correlation of rGP/R to changes in GP_max_ (**Fig 2**).

The comparison of NPQ_max_ values revealed the largest interspecies differences between *Chaetoceros* sp. and *P*. *antarctica* (**Fig 1D**). At all growth conditions, NPQ_max_ values in *Chaetoceros* sp. were significantly higher (1 and 4°C with p < 0.001; -1°C with p < 0.05) than in *P*. *antarctica*. In contrast, there was no significant influence of temperature or salinity on NPQ_max_ in neither *Chaetoceros* sp. nor in both *Phaeocystis* strains. The species-specific differences in NPQ_max_ were further supported by the ratio of the half-saturation irradiance of NPQ_max_ (*E*_50_) over the photoacclimation parameter *E*_k_ (derived from fluorescence-based photosynthetic rates P_F_). Thus, the ratio *E*_50_/*E*_k_ describes the light-dependent NPQ induction status in relation to the saturation level of the electron transport chain. It is evident that the mean value *E*_50_/*E*_k_ for all experimental conditions was significantly higher in *Chaetoceros* sp. (mean *E*_50_/*E*_k_ = 4.0) compared to both strains of *Phaeocystis* (mean *E*_50_/*E*_k_ = 2.0; **S3 Fig**).

**Fig 3:**
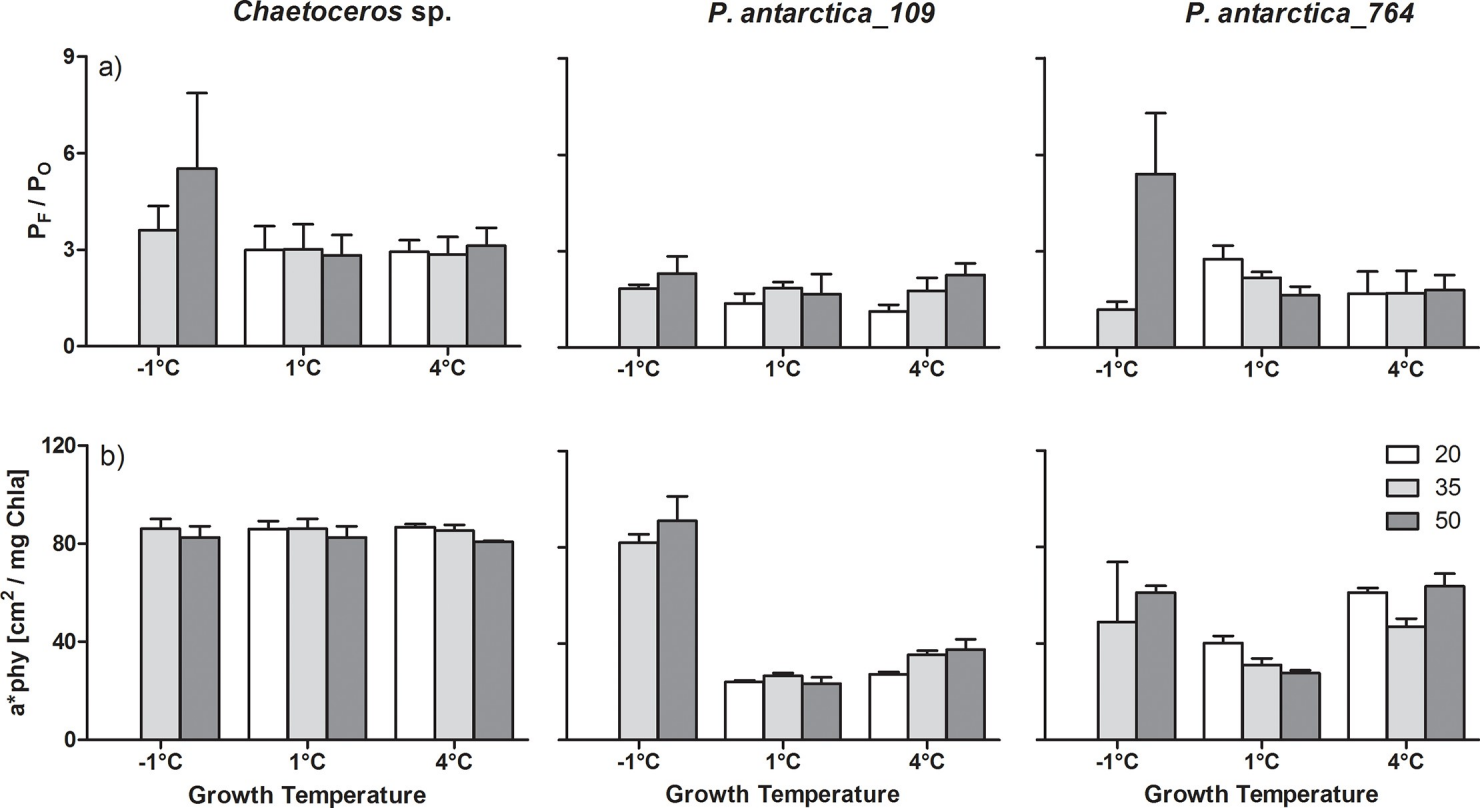
Physiological key parameters (P_F_/P_O_, a*_phy_) of *Chaetoceros* sp. and *Phaeocystis antarctica*. Mean values (± standard deviation) of physiological parameters measured in *Chaetoceros* sp. and *Phaeocystis antarctica* (strains 109 and 764) grown under different combinations of temperature (-1, 1, 4°C) and salinity of the growth medium (20, 35, 50, PSU; white, light grey, dark grey, respectively): a) Ratio maximum fluorescence-/maximum oxygen-based photosynthetic rate (P_F_/P_O_, n = 4–11), b) Chlorophyll-specific absorption coefficient (a*_phy_, [cm^2^ (mg Chl*a*)^-1^], n = 3). ‘n’ depicts the number of biological replicates. The same column colours with respect to medium salinity were applied for all subfigures.

The ratio of maximum fluorescence-based to maximum oxygen-based gross photosynthetic rates (P_F_/P_O_) is depicted in **Fig 3A**. For all investigated species no significant influence of temperature or salinity on P_F_/P_O_ was observed at growth temperatures of 1 and 4°C. Only at -1°C P_F_/P_O_ was significantly increased in *Chaetoceros* sp. and in *P*. *antarctica* strain 764 compared to 1 and 4°C, respectively (at 50 PSU; p < 0.001). As a consequence, P_F_/P_O_ was significantly lower at -1°C/50 PSU in *P*. *antarctica* strain 109 than in strain 764 (p < 0.01).

The mean value of the Chl-specific *in vivo*-absorption (a*_phy_) describes the absorption efficiency of algal cells. Under the experimental conditions, *Chaetoceros* sp. showed the lowest variation of a*_phy_ values with no significant influence of neither temperature nor salinity (**Fig 3B**). Similarly, there was no significant influence of salinity on the absorption efficiency of both strains of *P*. *antarctica*. On the other hand, in both strains of *P*. *antarctica* a large variation of a*_phy_ values was observed. Accordingly, at a growth temperature of 1°C and 4°C the a*_phy_ values were significantly lower in both strains of *P*. *antarctica* at all salinities than in *Chaetoceros* sp. (p < 0.001). In addition, *P*. *antarctica* strain 764 showed significantly higher a*_phy_ values than strain 109 at a growth temperature of 4°C. In contrast, at -1°C growth temperature a*_phy_ values were in a comparable range for all three algal species. Interestingly, this resulted in a specific pattern of a*_phy_ changes with respect to those experimental conditions that represent different seasonal conditions (**S4 Fig**; see **Table 1** for experimental conditions). Whereas *Chaetoceros* sp. showed constant a*_phy_ values over all seasonal conditions, in *P*. *antarctica* strain 109 significantly higher a*_phy_ values were observed in the winter condition than in the other seasonal conditions (p < 0.01). Thereby, strain 109 reached similar a*_phy_ values under winter conditions as *Chaetoceros* sp. In *P*. *antarctica* strain 764 significantly higher a*_phy_ was observed in the winter condition than in spring and autumn (p < 0.01).

### rGP/R and NPP under seasonal conditions

**Fig 4:**
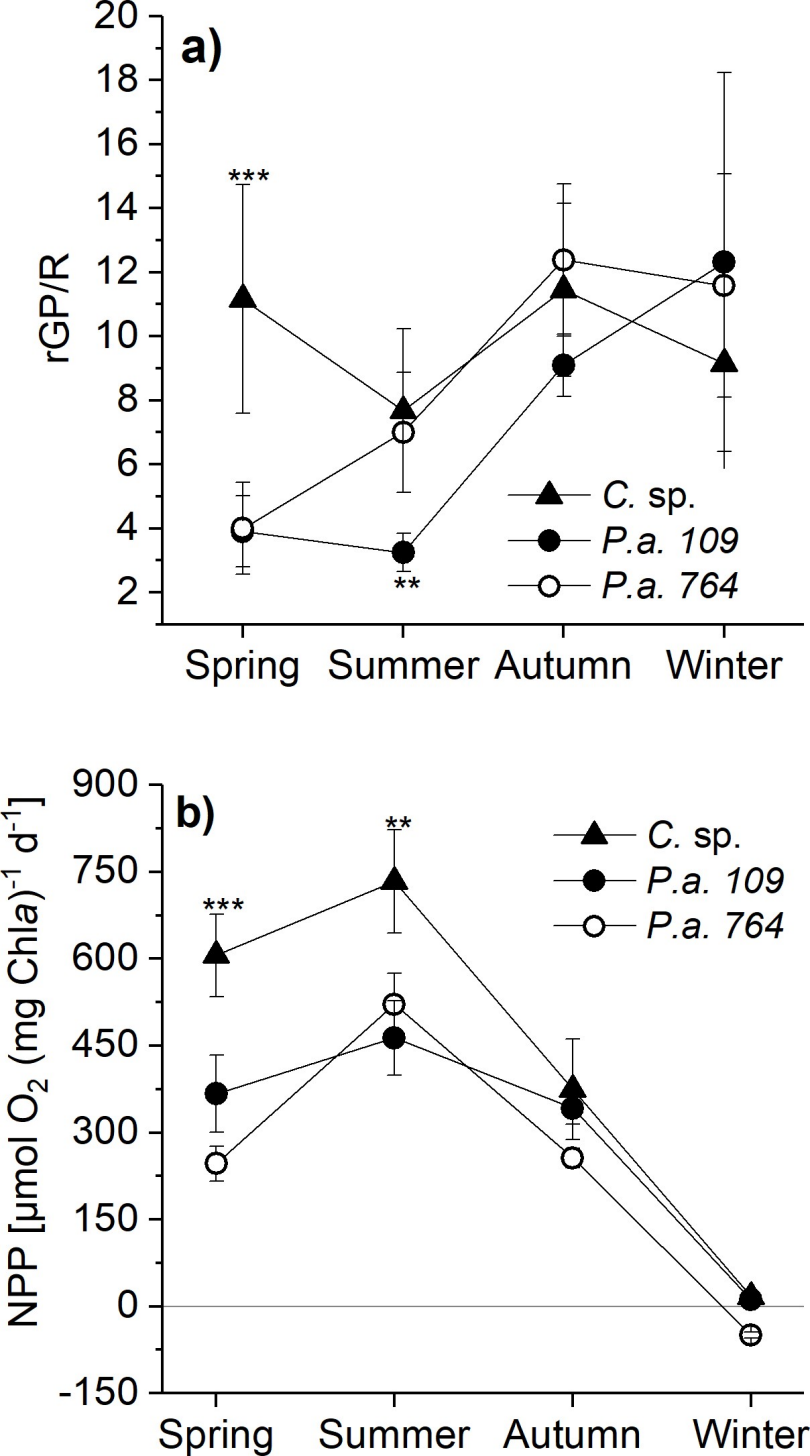
Ratio gross photosynthesis to respiration and calculated net primary production (NPP) for different seasonal conditions. The ratio P/R and NPP were derived from mean values (± standard deviation) measured in *Chaetoceros* sp. (*C*. sp., filled triangles), *Phaeocystis antarctica* (*P*.*a*., strains 764, open circles, and 109, filled circles) grown under experimental conditions that represent specific seasonal *in situ*-conditions: a) Spring, b) Summer, c) Autumn, d) Winter (see text for details). NPP (μmol O_2_ [mg Chl*a*]^-1^ d^-1^) was calculated from fitted gross oxygen production rates (GP) minus measured respiratory losses considering the light conditions under seasonal conditions. The asterisks represent significant differences between the species (* p < 0.05, ** p < 0.01, *** p < 0.001).

The large range of applied experimental conditions was chosen to investigate the general influence of salinity and temperature on the physiology of Antarctic phytoplankton. However, phytoplankton will not be confronted with all of these conditions in their natural environment. Therefore, **Fig 4A** depicts the changes in rGP/R under those experimental conditions that represent the salinity/temperature combinations of different seasonal conditions. Whereas, no significant changes of rGP/R under different seasonal conditions were observed in *Chaetoceros* sp., there was a significant increase of rGP/R from the spring/summer to the autumn/winter conditions (p < 0.05) in both strains of *P*. *antarctica*.

**Fig 5:**
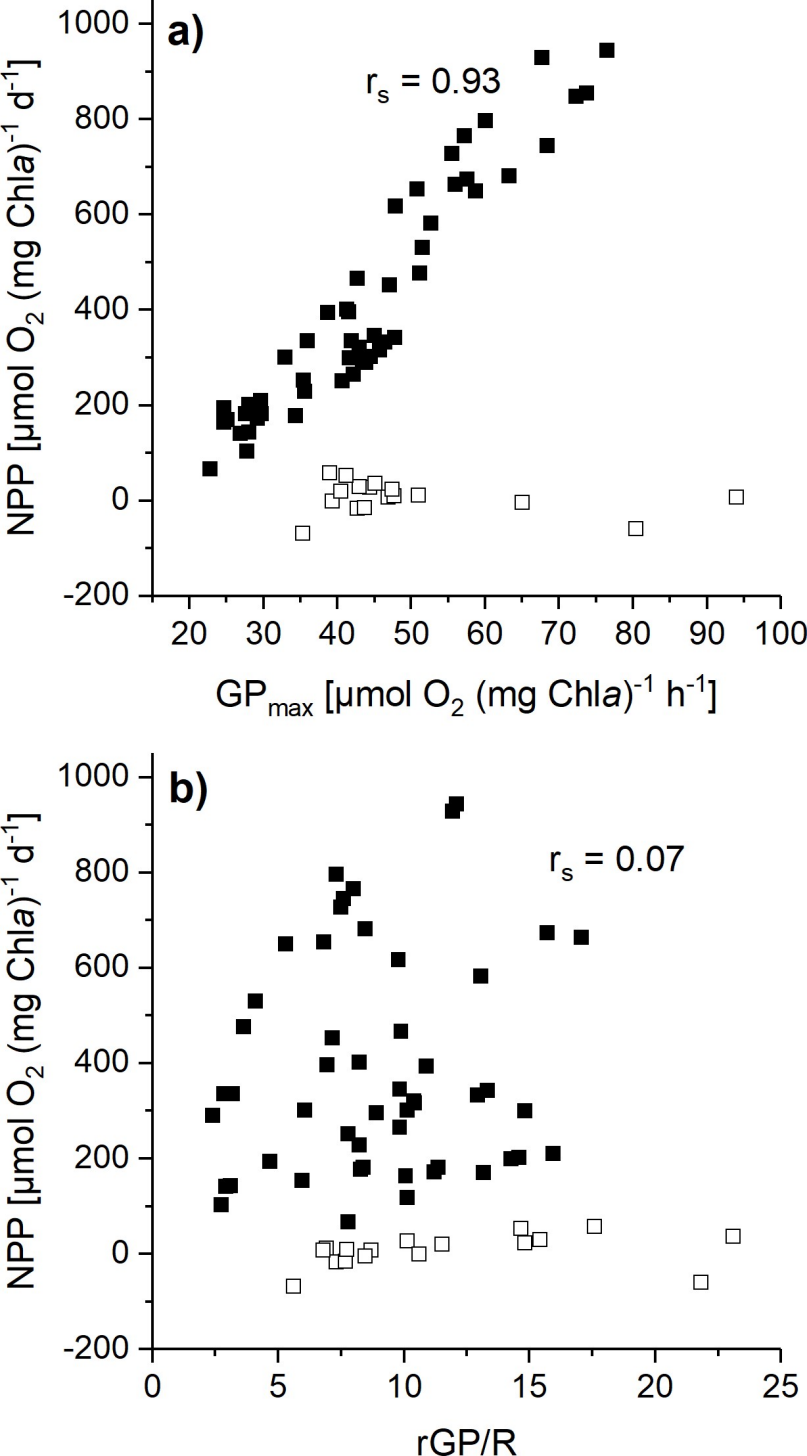
Relationship between calculated net primary production (NPP) and GP_max_ and between NPP and rGP/R. NPP (μmol O_2_ [mg Chl*a*]^-1^ d^-1^) was calculated from fitted gross oxygen production (GP) minus measured respiratory losses in *Chaetoceros* sp. (*C*. sp.), *Phaeocystis antarctica* (*P*.*a*., strains 764 and 109). Estimation of NPP is based on mean values of fitted Photosynthesis-Irradiance curves for different experimental conditions that represent specific seasonal *in situ*-conditions (see text for details). The calculated NPP values were plotted against the respective mean values of maximum gross photosynthesis (GP_max_) and against rGP/R, respectively. The correlation was calculated using Spearman rank correlation (correlation coefficient r_s_). In a), the correlation between NPP and GP_max_ data was calculated separately for ‘Spring’, ‘Summer’, and ‘Autumn’ (filled squares) and for the ‘Winter’ condition (open squares).

It was additionally intended to evaluate the effect of changes in rGP/R on NPP. For this purpose, daily-integrated NPP for different seasonal conditions (**Fig 4B**) were calculated on the basis of the measured photosynthesis and respiration rates under the specific experimental conditions in combination with season-specific *in situ* light conditions (see Materials & methods for details). As expected from seasonal *in situ* light conditions (with respect to maximum irradiance and daylength), the highest NPP was calculated for summer conditions, whereas under winter conditions a barely positive NPP was calculated for *Chaetoceros* sp. and *P*. *antarctica* strain 109, but not for strain 764. Although, there was the trend of higher rGP/R in autumn/winter than in spring/summer, **Fig 5** reveals that there is no correlation between rGP/R and NPP for different seasonal conditions. Instead, a significant, positive correlation between GP_max_ and NPP is found for the spring, summer, and autumn conditions (p < 0.01). For the winter condition, there is no correlation between GP_max_ and NPP.

## Discussion

### Effects of temperature and salinity on photosynthetic and respiration rates

Several studies highlighted the importance of phytoplankton on microbial respiration and gross carbon production in the SO [9]. However, to our knowledge there is no study dealing with the influence of multiple stressors on both, photosynthesis and respiration rates, in Antarctic phytoplankton. Thus, the present study focussed on the investigation of the ratio photosynthesis to respiration under different combinations of temperature and salinity in two typical phytoplankton species of the SO.

Accordingly, the analysis of photosynthesis rates revealed no clear trend of temperature or salinity-dependent changes in GP_max_ values in *Chaetoceros* sp. and *Phaeocystis antarctica*. Although this observation is in accordance with previous studies (e.g. [11,33]) it is rather unexpected because GP_max_ is mainly defined by the activity of the enzyme RubisCO, whose activity should be directly correlated with temperature changes. A possible explanation for this observation could be that the solubility of CO_2_ with decreasing temperature increases more than that of O_2_ [34] and that the temperature effect can be compensated by a higher cellular RubisCO content at lower temperature [35].

The present study further revealed that only *Chaetoceros* sp. but not *P*. *antarctica* was able to grow at a combination of -1°C and a salinity of 70 PSU. To our knowledge this was not shown before and it could explain that *P*. *antarctica* is usually found in younger sea ice with conditions comparable to the water column but is rarely found in older sea ice (with higher salinity) [36].

In contrast to photosynthetic rates, a general trend of increasing respiration rates with the increase of growth temperature from 1 to 4°C at a salinity of 35 PSU was observed in both strains, *Chaetoceros* sp. and *P*. *antarctica*. In *P*. *antarctica*, this trend was also found at a salinity of 50 PSU. These changes in respiration rates also influenced the temperature-dependent changes of the ratio of gross photosynthesis to respiration. At a salinity of 35 PSU, *Chaetoceros* sp. and both strains of *P*. *antarctica* showed decreasing rGP/R with increasing growth temperature from 1 to 4°C. From these results two major conclusions could be drawn: first, the changes in rGP/R were primarily due to variations in respiration but not in photosynthetic rates, and second, rGP/R is primarily temperature-dependent, whereas the impact of the salinity is of minor importance for rGP/R. The novel finding of the present study is that salinity influenced the temperature dependence of respiration to a very small degree in *Chaetoceros* sp., whereas in *P*. *antarctica* an effect of salinity was observed specifically in the combination with low salinity (20 PSU). Moreover, this study provides values of taxon-specific respiratory losses in SO phytoplankton. Accordingly, for all investigated experimental conditions, the respiratory losses in relation to GP were in the range of 8–14% in *Chaetoceros* sp., 8–25% in *P*. *antarctica* strain 764, and 8–33% in *P*. *antarctica* strain 109, with the lowest and highest losses at -1°C and 4°C, respectively. More specifically, *P*. *antarctica* showed significantly higher rGP/R values in autumn/winter compared to spring/summer whereas, the season-specific rGP/R values varied not significantly in *Chaetoceros* sp. In the light of these species-specific variation of rGP/R and of the observed species-specific temperature dependence of respiration it could be also concluded that the Q_10_ rule may not be systematically applicable in Antarctic phytoplankton.

### Respiratory losses and net primary production

An important aim of the present study was the evaluation of the impact of different rGP/R on NPP estimates in representative phytoplankton species from the SO. Therefore, the present data set was used to calculate NPP for specific irradiance, temperature, and salinity combinations that represent different seasonal conditions. The comparison of species-specific NPP for the different seasons showed a comparable pattern for all investigated species. The highest NPP was calculated for the ‘Summer’ condition with high irradiance, short dark period, and high water temperatures. Compared to the ‘Summer condition’, NPP calculated for ‘Spring’ and ‘Autumn’ ranged at 28% in *P*. *antarctica* strain 764 and between 60–80% in *Chaetoceros* sp. and *P*. *antarctica* strain 109 (**Fig 4B**). Despite the large season-specific differences in rGP/R, the comparison of the calculated NPP with season-specific rGP/R and GP_max_ values in the ‘Spring’, ‘Summer’, and ‘Autumn’ conditions, respectively, revealed that the NPP is clearly correlated with the photosynthetic potential of the investigated phytoplankton species but not with their respiratory losses. This does not hold true for the ‘Winter’ condition and could be due to its very short light period (6/18h, L/D). Here, a positive NPP was calculated for *Chaetoceros* sp. and *P*. *antarctica* strain 109, only. This means that these algal strains are able to keep respiratory losses at a minimum and to maintain the cells in an energetic balance during ‘Winter’ condition. This is in line with results of [33] where strongly reduced but still positive carbon uptake rates were measured under a combination of low irradiance (5 μmol m^-2^ s^-1^) and low temperature (-1.5°C) in the diatom *Chaetoceros*.

### Species-specific differences in the acclimation to variations in temperature and salinity

A distinctive species-specific difference in the acclimation to different temperature and salinity combinations is based on the observation of lower variations of some physiological parameters (R, rGP/R, a*_phy_, P_F_/P_O_) in *Chaetoceros* sp. than in *P*. *antarctica*. Obviously, cells of *Chaetoceros* sp. are able to cope with strongly changing temperature and salinity conditions within the range of their actual physiological capacity, whereas cells of *P*. *antarctica* were forced to specifically acclimate their physiological cell status according to the experimental conditions. This could be interpreted as different acclimation strategies of phytoplankton, which is also reflected by significantly higher NPQ_max_ values, higher rGP/R (at 1 and 4°C), higher P_F_/P_O_ (at 1 and 4°C), and a generally higher ratio *E*_50_/*E*_k_ in *Chaetoceros* sp. than in *P*. *antarctica*. The ratio *E*_50_/*E*_k_ describes the half-saturation light intensity of NPQ_max_ in relation to the beginning saturation of photosynthetic rates. The significantly higher *E*_50_/*E*_k_ in *Chaetoceros* sp. could be interpreted in the way that the full potential of light protection in the investigated species was required at very high irradiance only, which is an indication of a very high overall potential of light protection. Thus, in our opinion, the higher NPQ_max_ values in *Chaetoceros* sp. compared to *P*. *antarctica* are not an indication of photoinhibitory stress but of a high photoprotective potential. The species dependence of NPQ_max_ values in the comparison of different Antarctic phytoplankton species and, in particular, the higher NPQ_max_ in diatoms than NPQ_max_ in *P*. *antarctica* was also shown in previous publications [18,37,38]. The non-photochemical quenching is designated as a very important mechanism to adapt to dynamic light conditions as experienced by the phytoplankton in their natural habitats (e.g. [39]). The most important component of NPQ is the energy-dependent quenching that depends on the presence of a proton gradient across the thylakoid membrane, of de-epoxidized xanthophyll cycle pigments, and of specific light-harvesting proteins (Lhcx) [40]. It is therefore likely that the higher NPQ capacity in *Chaetoceros* sp. than in *P*. *antarctica* is due to a larger pool size of xanthophyll cycle pigments and/or to an increased Lhcx protein content of the cells [41].

With respect to the photoprotective potential of phytoplankton, the extent of alternative electron transport is of importance. The significantly higher ratio P_F_/P_O_ in *Chaetoceros* sp. than in *P*. *antarctica* (at 1 and 4°C with 35 PSU) could be interpreted as a higher activity of alternative electron pathways [10]. Alternative electrons are not used for the reduction of NADP^+^. Instead, they contribute to e.g. cyclic electron transport at PSII and PSI, to the water-water cycle, to photorespiration, to the reduction of nitrate and sulphate [42,43] and, thus, to the generation of the trans-thylakoid pH gradient. Therefore, it is assumed that the activity of alternative electron transport changes the photosynthetic NADPH/ATP ratio in favour of ATP which in turn decreases the energetic pressure on the photosynthetic electron transport chain [27]. It is not known whether this additional ATP production could compensate for ATP production by e.g. lower respiration rates. However, notably high P_F_/P_O_ values were observed in *Chaetoceros* sp. and *P*. *antarctica* strain 764 at the ‘Winter’ condition (-1°C, 50 PSU) where at the same time low relative respiratory losses were measured. Thus, alternative electron sinks could contribute to dissipate excessively absorbed light energy to maintain the cellular energy balance under unfavourable conditions [42].

The different acclimation strategy to changing temperature and salinity conditions could be also deduced from the comparison of a*_phy_ values. Whereas a*_phy_ did not vary significantly in *Chaetoceros* sp., a significant increase of a*_phy_ in both strains of *P*. *antarctica* was observed in the ‘Winter’ condition (**S4 Fig**). The a*_phy_ value describes the wavelength-dependent and Chl*a*-normalized absorptivity (spectrally integrated optical absorption cross section) of phytoplankton cells. It depends to a large extent on cellular Chl concentration and the related package effect of pigments but also on the content of accessory pigments. Thus, an increase of a*_phy_ is typically induced by a decrease of cellular Chl content [44]. It could be concluded that the cellular Chl content did not change in *Chaetoceros* sp. under the applied experimental conditions whereas the results indicate a decreased cellular Chl content in *P*. *antarctica* at low temperature in combination with high salinity.

In summary, the species-specific differences observed in the present study might reflect the specific adaptation of Antarctic phytoplankton to different environmental conditions, e.g. to sea ice or highly stratified water conditions in the case of *Chaetoceros* sp., in contrast to deeply mixed waters in the pelagic zone in the case of *P*. *antarctica* [6].

## Conclusions

In the light of the importance of the SO for the atmospheric CO_2_ level, it is essential to understand the influence of combined changes of environmental factors on respiratory losses in relation to the photosynthetic activity of the phytoplankton. The present study on two different species of Antarctic algae has shown that particularly temperature changes induce variations of rGP/R. However, these variations did not influence NPP. It could be therefore concluded that the assumption of constant respiratory loss rates in the range of 10–15% of GP within the annual growth period appears appropriate under field conditions when measured respiration data are not available. It should be emphasized that changes of other environmental factors (e.g. nutrient availability, grazing pressure) may induce stronger variation of rGP/R. In this case, the impact on NPP needs to be re-evaluated.

## Supporting information

S1 Table Temperature and salinity of the applied experimental conditions and numbers of biological replicates for the measured parameters. The numbers in the table represent the numbers of biological replicates for the measured physiological parameters under the applied experimental conditions: GP_max_, maximum gross photosynthetic rate; R, respiration rate; rGP/R, ratio of maximum gross photosynthetic rate to respiration rate; NPQ, non-photochemical quenching; P_F_/P_O_, ratio fluorescence-based to oxygen-based gross photosynthetic rate; a*_phy_, Chlorophyll-specific absorption coefficient.
(DOCX)

S1 Fig Representative example of measurements of photosynthesis rates and non-photochemical quenching (NPQ). In a) oxygen-based net photosynthetic rates (P_O_; μmol O_2_ [mg Chl*a*]^-1^ h^-1^) as function of irradiance in *Chaetoceros* sp. (filled triangles) and *Phaeocystis antarctica* (strain 109; filled circles) grown at 4°C and 35 PSU are depicted. Dotted lines show the fitted photosynthetic-irradiance curves of *Chaetoceros* sp. and *P*. *antarctica*, respectively. In b) the fluorescence-based gross photosynthetic rates (P_F_; μmol O_2_ [mg Chl*a*]^-1^ h^-1^) as function of irradiance in *Chaetoceros* sp. and *P*. *antarctica* are depicted. c) Light-dependent increase of non-photochemical quenching (NPQ; [Fm-Fm’]/Fm’) in *Chaetoceros* sp. and *P*. *antarctica*.
(DOCX)

S2 Fig Light conditions used for the estimation of daily net primary production from measured photosynthesis and respiration rates. Light conditions were adopted from Petrou & Ralph (2011) and represent *in situ* irradiance (PAR, photosynthetically available radiation) for phytoplankton in summer (Pelagic), autumn (New sea ice), winter (Sea ice), and spring (Meltwater).
(DOCX)

S3 Fig Ratio of half-saturation irradiance of maximum NPQ (*E*_50_) over characteristic irradiance *E*_k_ derived from fluorescence-based photosynthetic-irradiance curves in *Chaetoceros* sp. (*C*. sp.), *Phaeocystis antarctica* strain 764 (*P*.*a*. 764) and *P*. *antarctica* strain 109 (*P*.*a*. 109). Data represent means of all experimental conditions (n = 9). The level of significance is indicated by *** (p < 0.001).
(DOCX)

S4 Fig The Chl-specific absorption a*_phy_ was derived from mean values (± standard deviation) measured in *Chaetoceros* sp. (*C*. sp., filled triangles), *Phaeocystis antarctica* (P.a., strains 764, open circles, and 109, filled circles) grown under experimental conditions that represent specific seasonal in situ-conditions: Spring, Summer, Autumn, Winter. The level of significance between the species is indicated by ** (p < 0.01), *** (p < 0.001).
(DOCX)
