# Supplementary material for: Effects of temperature and salinity on respiratory losses and the ratio of photosynthesis to respiration in representative Antarctic phytoplankton species
Source: PLoS One. 2019 Oct 21;14(10):e0224101. doi: 10.1371/journal.pone.0224101 (PMC6802872; doi:10.1371/journal.pone.0224101)

**Supporting material Bozzato et al.**

**Supporting Table 1. Temperature and salinity of the applied experimental conditions and numbers of biological replicates for the measured parameters.** The numbers in the table represent the numbers of biological replicates for the measured physiological parameters under the applied experimental conditions: GP_max_, maximum gross photosynthesis rate; R, respiration rate; rGP/R, ratio of maximum gross photosynthesis rate to respiration rate; NPQ, non-photochemical quenching; P_F_/P_O_, ratio fluorescence-based to oxygen-based gross photosynthesis rate; *a**_phy_, Chlorophyll-specific absorption coefficient.


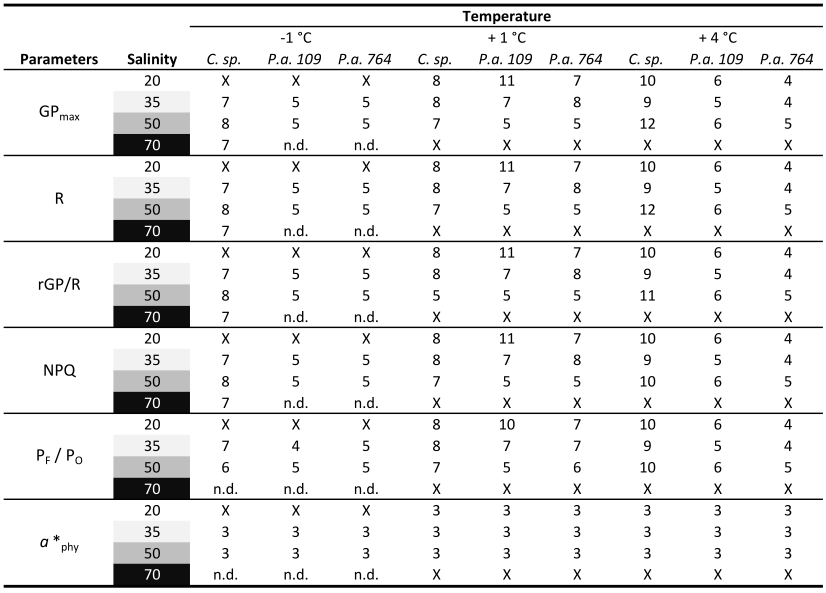

Supplement: S1 Table — The numbers in the table represent the numbers of biological replicates for the measured physiological parameters under the applied experimental conditions: GPmax, maximum gross photosynthetic rate; R, respiration rate; rGP/R, ratio of maximum gross photosynthetic rate to respiration rate; NPQ, non-photochemical quenching; PF/PO, ratio fluorescence-based to oxygen-based gross photosynthetic rate; a*phy, Chlorophyll-specific absorption coefficient. (DOCX) [file pone.0224101.s001.docx]
