# Supplementary material for: Effects of temperature and salinity on respiratory losses and the ratio of photosynthesis to respiration in representative Antarctic phytoplankton species
Source: PLoS One. 2019 Oct 21;14(10):e0224101. doi: 10.1371/journal.pone.0224101 (PMC6802872; doi:10.1371/journal.pone.0224101)
Supplement: S1 Fig — In a) oxygen-based net photosynthetic rates (PO; μmol O2 [mg Chla]-1 h-1) as function of irradiance in Chaetoceros sp. (filled triangles) and Phaeocystis antarctica (strain 109; filled circles) grown at 4°C and 35 PSU are depicted. Dotted lines show the fitted photosynthetic-irradiance curves of Chaetoceros sp. and P. antarctica, respectively. In b) the fluorescence-based gross photosynthetic rates (PF; μmol O2 [mg Chla]-1 h-1) as function of irradiance in Chaetoceros sp. and P. antarctica are depicted. c) Light-dependent increase of non-photochemical quenching (NPQ; [Fm-Fm’]/Fm’) in Chaetoceros sp. and P. antarctica. (DOCX) [file pone.0224101.s002.docx]

**Supporting Fig 1. Representative example of measurements of photosynthesis rates and non-photochemical quenching (NPQ).** In a) oxygen-based net photosynthesis rates (P_O_; µmol O_2_ [mg Chl*a*]^-1^ h^-1^) as function of irradiance in *Chaetoceros* sp. (filled triangles) and *Phaeocystis antarctica* (strain 109; filled circles) grown at 4°C and 35 PSU are depicted. Dotted lines show the fitted photosynthesis-irradiance curves of *Chaetoceros* sp. and *P. antarctica*, respectively. In b) the fluorescence-based gross photosynthesis rates (P_F_; µmol O_2_ [mg Chl*a*]^-1^ h^-1^) as function of irradiance in *Chaetoceros* sp. and *P. antarctica* are depicted. c) Light-dependent increase of non-photochemical quenching (NPQ; [Fm-Fm’]/Fm’) in *Chaetoceros* sp. and *P. antarctica.*

**

**
