# Supplementary material for: Effects of temperature and salinity on respiratory losses and the ratio of photosynthesis to respiration in representative Antarctic phytoplankton species
Source: PLoS One. 2019 Oct 21;14(10):e0224101. doi: 10.1371/journal.pone.0224101 (PMC6802872; doi:10.1371/journal.pone.0224101)

**Supporting material Bozzato et al.**

**Supporting Fig 2.** **Light conditions used for the estimation of daily net primary production from measured photosynthesis and respiration rates.** Light conditions were adopted from Petrou & Ralph (2011) and represent *in situ* irradiance (PAR, photosynthetically available radiation) for phytoplankton in summer (Pelagic), autumn (New sea ice), winter (Sea ice), and spring (Meltwater).


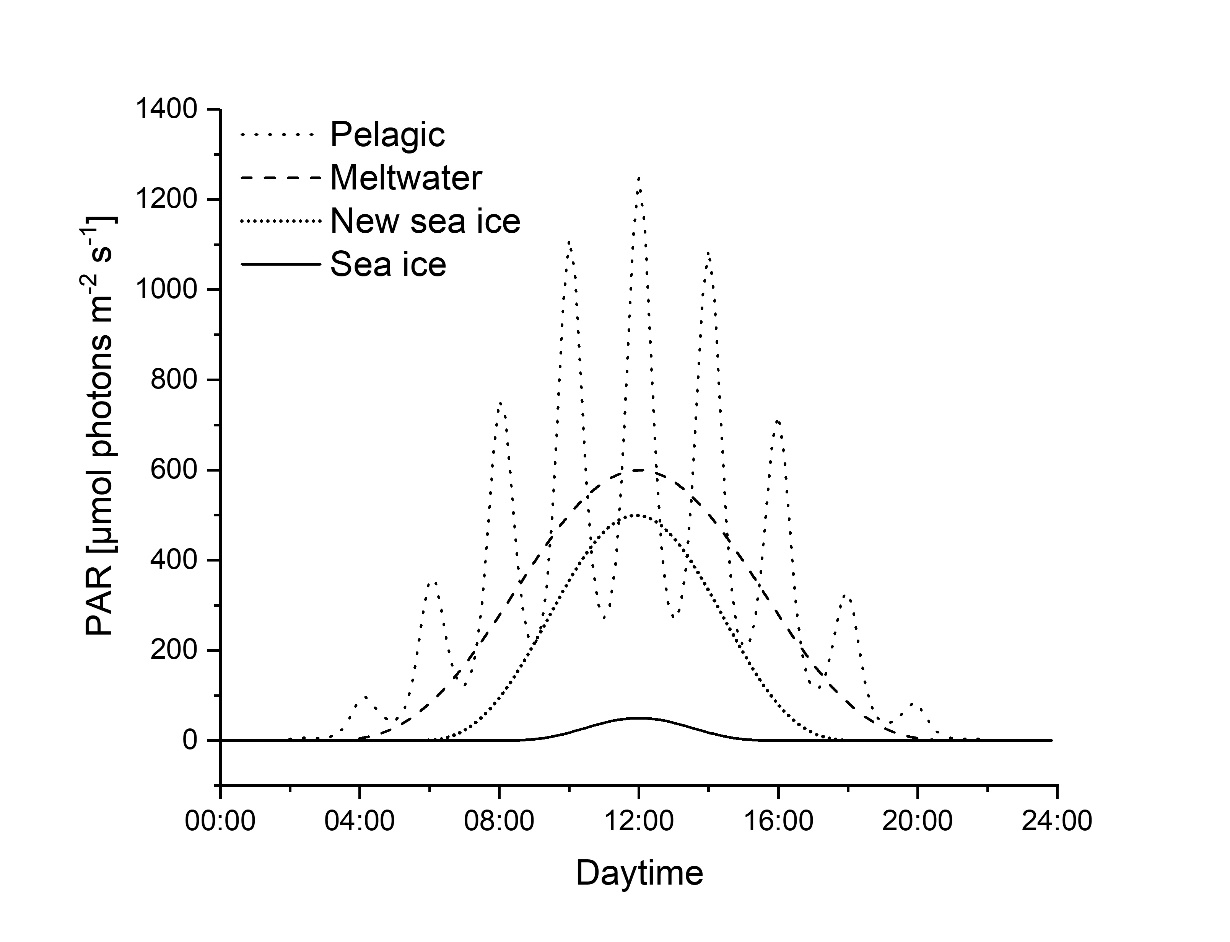

Supplement: S2 Fig — Light conditions were adopted from Petrou & Ralph (2011) and represent in situ irradiance (PAR, photosynthetically available radiation) for phytoplankton in summer (Pelagic), autumn (New sea ice), winter (Sea ice), and spring (Meltwater). (DOCX) [file pone.0224101.s003.docx]
