# Supplementary material for: Effects of temperature and salinity on respiratory losses and the ratio of photosynthesis to respiration in representative Antarctic phytoplankton species
Source: PLoS One. 2019 Oct 21;14(10):e0224101. doi: 10.1371/journal.pone.0224101 (PMC6802872; doi:10.1371/journal.pone.0224101)
Supplement: S3 Fig — Data represent means of all experimental conditions (n = 9). The level of significance is indicated by *** (p < 0.001). (DOCX) [file pone.0224101.s004.docx]

**Supporting material Bozzato et al.**

**Supporting Fig 3:** Ratio of half-saturation irradiance of maximum NPQ (*E*_50_) over characteristic irradiance *E*_k_ derived from fluorescence-based photosynthesis-irradiance curves in *Chaetoceros* sp. (*C*. sp.), *Phaeocystis antarctica* strain 764 (*P.a.* 764) and *P. antarctica* strain 109 (*P.a.* 109). Data represent means of all experimental conditions (n = 9). The level of significance is indicated by *** (p < 0.001).
