# Supplementary material for: Effects of temperature and salinity on respiratory losses and the ratio of photosynthesis to respiration in representative Antarctic phytoplankton species
Source: PLoS One. 2019 Oct 21;14(10):e0224101. doi: 10.1371/journal.pone.0224101 (PMC6802872; doi:10.1371/journal.pone.0224101)
Supplement: S4 Fig — The level of significance between the species is indicated by ** (p < 0.01), *** (p < 0.001). (DOCX) [file pone.0224101.s005.docx]

**Supporting material Bozzato et al.**

**Supporting Fig 4:** The Chl-specific absorption a*_phy_ was derived from mean values (± sd) measured in *Chaetoceros* sp. (*C.* sp., filled triangles), *Phaeocystis antarctica* (*P.a.*, strains 764, open circles, and 109, filled circles) grown under experimental conditions that represent specific seasonal *in situ*-conditions: a) Spring, b) Summer, c) Autumn, d) Winter. The level of significance between the species is indicated by ** (p < 0.01), *** (p < 0.001).
